# Acoustic cues for the recognition of self-voice and other-voice

**DOI:** 10.3389/fpsyg.2013.00735

**Published:** 2013-10-11

**Authors:** Mingdi Xu, Fumitaka Homae, Ryu-ichiro Hashimoto, Hiroko Hagiwara

**Affiliations:** Department of Language Sciences, Graduate School of Humanities, Tokyo Metropolitan UniversityTokyo, Japan

**Keywords:** self recognition, voice recognition, speech perception, fundamental frequency, formant

## Abstract

Self-recognition, being indispensable for successful social communication, has become a major focus in current social neuroscience. The physical aspects of the self are most typically manifested in the face and voice. Compared with the wealth of studies on self-face recognition, self-voice recognition (SVR) has not gained much attention. Converging evidence has suggested that the fundamental frequency (F0) and formant structures serve as the key acoustic cues for other-voice recognition (OVR). However, little is known about which, and how, acoustic cues are utilized for SVR as opposed to OVR. To address this question, we independently manipulated the F0 and formant information of recorded voices and investigated their contributions to SVR and OVR. Japanese participants were presented with recorded vocal stimuli and were asked to identify the speaker—either themselves or one of their peers. Six groups of 5 peers of the same sex participated in the study. Under conditions where the formant information was fully preserved and where only the frequencies lower than the third formant (F3) were retained, accuracies of SVR deteriorated significantly with the modulation of the F0, and the results were comparable for OVR. By contrast, under a condition where only the frequencies higher than F3 were retained, the accuracy of SVR was significantly higher than that of OVR throughout the range of F0 modulations, and the F0 scarcely affected the accuracies of SVR and OVR. Our results indicate that while both F0 and formant information are involved in SVR, as well as in OVR, the advantage of SVR is manifested only when major formant information for speech intelligibility is absent. These findings imply the robustness of self-voice representation, possibly by virtue of auditory familiarity and other factors such as its association with motor/articulatory representation.

## Introduction

The concept of “self” has attracted people interested in diverse fields from philosophy and literature to neuroscience. Self-recognition (the capacity to recognize physical and mental aspects of oneself) is a highly developed ability in humans that underlies a range of social and interpersonal functions, such as the theory of mind and introspection (Gallup, 1982, 1985; Rosa et al., 2008). Recent social neuroscience studies have made considerable progress in identifying neural mechanisms underlying various types of self-related information processing. The majority of such studies targeted self-face recognition, because self-face is considered the actual embodiment of self-image (representation of one's own identity) (Uddin et al., 2007; Kaplan et al., 2008). Although not as effective as face recognition, humans also possess the ability to recognize voices without seeing the speakers' faces; for example, while talking to someone over the telephone. Individual speech, regarded as the “auditory face,” conveys a wealth of socially relevant paralinguistic information (e.g., physical/emotional state) (Nakamura et al., 2001; Belin et al., 2002, 2004; Yovel and Belin, 2013). Self-voice recognition (SVR) is extremely important, since it is essential for self-consciousness and self-monitoring during speech production. Its disruption can have a detrimental impact on mental health and can negatively affect one's quality of life (Ford and Mathalon, 2005; Johns et al., 2006; Allen et al., 2007; Asai and Tanno, 2013).

Accumulating neuroscientific evidence has revealed the temporal and spatial profiles of self-recognition in several sensory domains, especially self-face recognition (Ninomiya et al., 1998; Sugiura et al., 2005; Uddin et al., 2005). Recently, an event-related potential (ERP) study has reported that self-face recognition took place earlier than familiar other-face recognition in the brain and displayed more robust brain activity (Keyes et al., 2010). Another functional neuroimaging study has found that the right inferior frontal gyrus (IFG) consistently showed activation in response to both self-face and self-voice, suggesting its contribution to the abstract multimodal self-representation (Kaplan et al., 2008). Uddin et al. (2007) has put forward a notable proposal that the right-lateralized mirror-neuron system processes the perceived self, including both self-face and self-voice. Thus, all of these studies suggest the distinctiveness of self-related information processing, and that it is at least partially different from other-related information processing. In contrast to the gradual clarification of self-face recognition, however, the cues and mechanisms involved in SVR remain to be elucidated. To our knowledge, few behavioral studies have successfully clarified the acoustic cues for SVR, even though these cues may play a crucial role in differentiating between self-voice and other-voice.

Although people rarely utter words in an identical way, the variations in one's voice are generally around a mean “voice signature,” which determines one's vocal characteristics, allowing the listeners to remember and recognize the voice in the future (Belin et al., 2011). Moreover, the unique features in vocal signals are mostly attributable to the anatomical structures of one's articulatory system, as well as one's specific ways of using the organs of articulation (Hecker, 1971; Bricker and Pruzansky, 1976).

Specifically, as the source, the vocal folds in the larynx vibrate periodically with a well-defined fundamental frequency (F0; i.e., the perceived pitch), the average of which largely depends on the length and mass of one's vocal folds (Ghazanfar and Rendall, 2008). Previous studies have suggested that listeners rely on the average F0 to a large extent to discriminate and recognize different voices (Baumann and Belin, 2010; Chhabra et al., 2012). On the other hand, the vocal tract above the larynx functions as a filter. It allows the acoustic energy of the source signal that shares with it the same frequency (formant frequency) to pass through it, but obstructs the energy at other frequencies (Ghazanfar and Rendall, 2008). The frequencies of formants are considered to be related to both the size of one's vocal tract and the particular gestures of one's articulatory apparatus during speech (Ghazanfar and Rendall, 2008; Latinus and Belin, 2011). The formant structures contribute to the unique perceived vocal timbre of a specific person, thus providing identity information (Remez et al., 1997; Ghazanfar and Rendall, 2008; Baumann and Belin, 2010; Macdonald et al., 2012). In particular, frequencies higher than 2500 Hz (typically including formants higher than the third formant, i.e., F3, of adults) are greatly related to the anatomy of one's laryngeal cavity, whose anatomical configuration varies between speakers but virtually remains unchanged during one's articulation of different vowels, and therefore carry some individual specificity (Dang and Honda, 1997; Kitamura et al., 2005, 2006; Takemoto et al., 2006). Furthermore, recent studies have suggested that both the F0 and formant structures play significant roles in outlining the individuality of other-voice (Rendall et al., 2005; Latinus and Belin, 2012).

It could be argued that the presence of bone conduction (Tonndorf, 1972; Maurer and Landis, 1990) is a methodological obstacle responsible for delayed progress in the study of SVR as compared with that of self-face perception. However, a similar but less severe difficulty in using self-stimuli exists even in the standard approaches of presenting photos of one's own faces because individuals may be more likely to recognize slightly morphed versions of their own faces, rather than their actual photos, as their own (Epley and Whitchurch, 2008). Since individuals are typically exposed to their own photos and voice recordings in numerous occasions in modern life, it has been suggested that both of these represent valid and appropriate self-stimuli for investigating self-perception of face and voice (Hughes and Nicholson, 2010).

Considering the uniqueness of self-related information, it is plausible that we use these acoustic cues differently in recognizing voices of our own versus others'. On the basis of such previous knowledge, the present study aimed to investigate the contributions of the F0 and formant structures to SVR. We predicted that (1) the more severe the modulation of F0, the lower the performance as long as the F0 information is available; however, when the F0 information is filtered out, there would be no effect of F0 modulation on performance; (2) when formants lower than F3, which determine the vowels, are available, people may recognize self-voice as well as other-voice; and (3) when neither F0 nor formants lower than F3 is accessible, performance might be higher for recognizing self-voice than other-voice, since the advantage of self-information, if any, would become apparent, particularly under situations in which acoustic cues carrying some individual specificity are highlighted.

## Materials and methods

### Participants

Six 5-person groups participated in the experiment—3 groups of females and 3 groups of males (mean age = 23.1 years, *SD* = 4.0 years). None of them reported any history of psychiatric or auditory illness. Within each group, the 5 members were academic colleagues, who knew each other's voice well. After reading a complete paper-based description of the study and receiving verbal instructions for the experiment, all the participants gave written informed consent to participate in the study, which was approved by the Human Subjects Ethics Committee of Tokyo Metropolitan University.

### Stimuli

**Figure 1:**
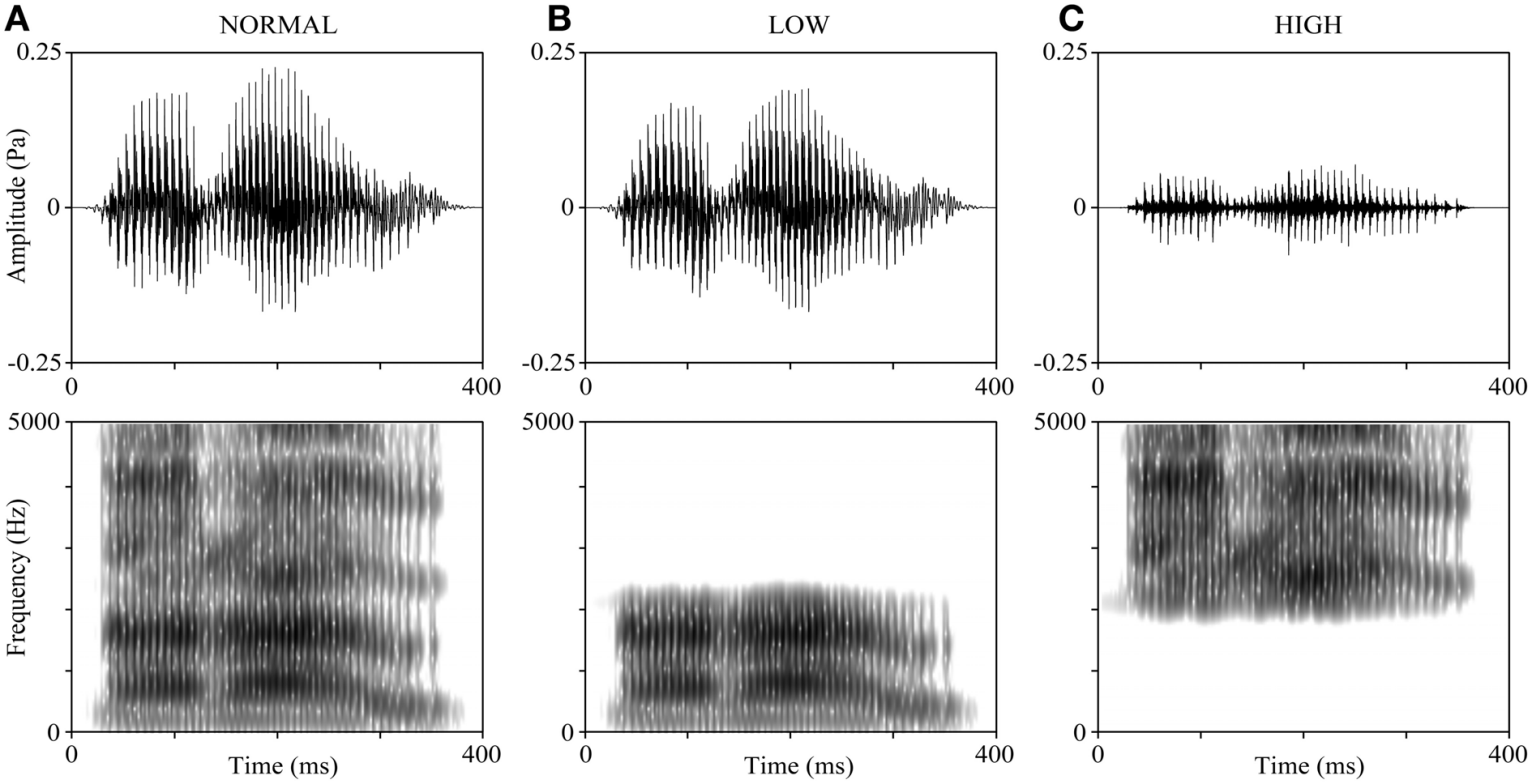
**Manipulations of the frequency band of speech sounds.** The voice sample is derived from a 20-year-old male participant reading “narau” (learn). **(A)** The original voice (NORMAL). **(B)** The LOW condition, where the voice was low-pass filtered at the cut-off frequency of the mean of F2 and F3. **(C)** The HIGH condition, where the voice was high-pass filtered at the cut-off frequency of the mean of F2 and F3. The upper panels display the waveforms and the lower panels display the spectrograms. The preceding and ending unvoiced parts (about 200 ms) are not shown in the figure.

The voices of all participants reading 5 Japanese sentences and 4 Japanese verbs were recorded before (1–2 weeks) the experiment. All the verbs are 3 morae long (the mora is a phonological unit in Japanese) and emotionally neutral: “ikiru” (live), “kimeru” (decide), “narau” (learn), and “todoku” (arrive). The participants were requested to pronounce the verbs clearly in Tokyo dialect. They were also asked to control their reading speed to be similar to a standardized sample recording as much as possible; thus, the duration of all verb-reading stimuli was around 600 ms. The voices were recorded using CoolEdit2000 (Adobe, Inc., San Jose, CA) at a sampling rate of 44.1 kHz and saved as WAV files. Figure 1A shows an example of the waveform and spectrogram of the verb “narau” (learn) read by a 20-year-old male. The mean F0 of recorded voices was 142.19 ± 20.97 Hz for the male group and 235.67 ± 24.89 Hz for the female group. We created 15 variations from each word read by each participant, by manipulating the F0 and the frequency bands. First, we shifted values of the F0 throughout the interval of the vocal stimuli of each word and made 5 types of variations (−4, −2, 0, +2, and +4 semitones), where “0” represents no manipulation, “+” represents raising the F0 values, and “−” represents lowering the F0 values. Shifts of 2 semitones (moderate modulation) and 4 semitones (severe modulation) in the F0 values were used because they made the speaker's voice more difficult to be recognized without making the speech incomprehensible (Allen et al., 2004; Johns et al., 2006). Next, 3 types of manipulations were applied to the frequency bands (NORMAL, LOW, and HIGH, as shown in Figures 1A–C). For the NORMAL condition, no frequency band manipulation was applied; for the LOW and HIGH conditions, either only the lower frequencies or only the higher frequencies were retained using a cut-off frequency of the mean value of the second (F2) and third (F3) formants (Mean ± SD: 2298 ± 126 Hz for the 30 participants). Finally, the mean intensity of each stimulus (5 by 3 variations) was adjusted to 65 dB. All the above-mentioned manipulations on voices were performed with the Praat 5.3 software (University of Amsterdam).

### Task and procedure

Before starting the experiment, the participants listened to the recorded sentences read by both themselves and their colleagues to confirm the voices of the 5 members in the group. Then, the requirement of the task was introduced to them via short-term exercises using the recorded verbs, which were different from those used in the experiment. The participants were instructed that both the pitch and the acoustic characteristics of the voices would have been altered, thus directing their attention away from strategically focusing on any particular acoustic cue. In the experiment, the participants sat about 60 cm away from the loud speakers, and they were asked to listen to the vocal stimuli with their eyes closed for concentration and to identify the speaker, who would be either themselves or 1 of their 4 colleagues, as quickly and accurately as possible by vocal naming. They were asked for a forced-choice response for each stimulus, with a response limit of 5 s. For each participant, a total of 300 valid trials (15 variations of 4 words spoken by 5 persons as described above and thus, 20 trials for each of the 15 conditions) and 200 filler trials (e.g., temporally reversed versions of filtered voices) were separated into 10 blocks (50 trials in each block; duration, 250 s/block) with self-paced breaks between blocks. The numbers of trials using each of the 5 persons' voices were equal (20% for each person), but this was not told to the participants, in order to prevent them from balancing their answers. The trial sequence in each block was pseudo-randomized, with the constraint of no more than 3 consecutive trials of a specific word read by a specific person. The presentation of the vocal stimuli was controlled via the STIM2 (Neuroscan, Charlotte, NC) stimulus presentation software. The whole experiment took approximately 1 h.

### Data analysis

Task performance was examined in terms of accuracy rate, which was calculated as the percentage of the number of trials where the subjects correctly named the speaker. To elucidate the main purpose of the present study, i.e., how do the F0 and frequency bands influence SVR and other-voice recognition (OVR), grand-averaged accuracies were calculated separately for SVR and OVR. Repeated-measures analyses of variance (ANOVAs) were used for statistical analyses. The accuracy data of the 30 subjects in the 15 conditions were first submitted to three-way repeated-measures ANOVAs. The within-subject factors were Identity (2 levels: SVR, OVR), F0 (5 levels: −4, −2, 0, +2, +4 semitones), and Frequency Band (3 levels: NORMAL, LOW, HIGH). When necessary, Greenhouse-Geisser adjustment was applied in case of sphericity violations. Uncorrected degrees of freedoms, but corrected *p* values were used in the results description. For *post-hoc* comparisons, Bonferroni-corrected pair-wise contrasts were used.

## Results

**Figure 2:**
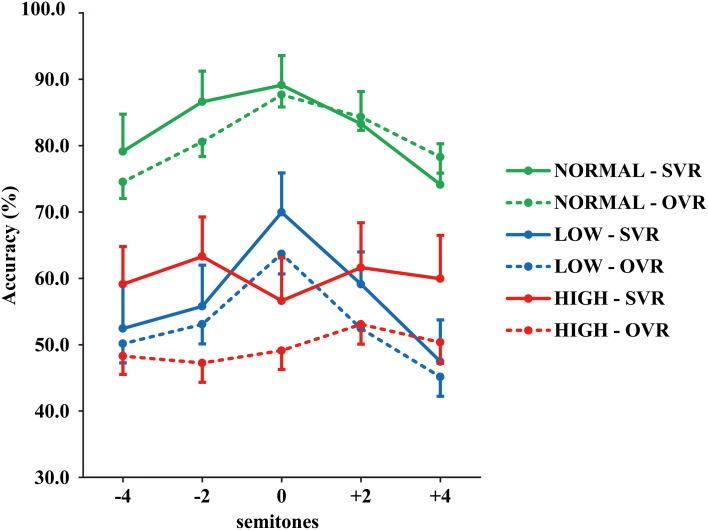
**Grand-averaged accuracies of SVR and OVR under the 15 conditions.** The accuracies for SVR are represented by solid lines and those for OVR are represented by broken lines. The green lines: NORMAL. The blue lines: LOW. The red lines: HIGH. The error bars represent the standard error of the mean among participants.

The grand-averaged accuracies of SVR and OVR under the 15 conditions are shown in Figure 2. (1) Accuracies of SVR and OVR decreased to a large degree in LOW and HIGH than in NORMAL. (2) In NORMAL and LOW, the accuracies of both SVR and OVR showed a clear “inverted U shape” with the peak at “0” as a function of F0 modulation. By comparison, the accuracies of SVR and OVR were relatively stable over the range of F0 modulation in HIGH. (3) Notably, in NORMAL and LOW, only a very minor difference was observed between the accuracy of SVR and OVR. In contrast, in HIGH, the accuracy of SVR was much higher than that of OVR.

Statistical analyses confirmed the above-mentioned observations. Three-way repeated-measures ANOVAs (Identity × F0 × Frequency Band) revealed significant main effects of both F0 [*F*_(4, 116)_ = 6.53, *p* < 0.001, η^2^_*p*_ = 0.18] and Frequency Band [*F*_(2, 58)_ = 93.61, *p* < 0.001, η^2^_*p*_ = 0.76], as well as a significant interaction of F0 × Frequency Band [*F*_(8, 232)_ = 5.30, *p* < 0.001, η^2^_*p*_ = 0.16]; all the other main effects and interactions not reported here failed to reach significance (all *p* > 0.1) (see Table 1). The effects of the F0 and Frequency Band were further examined using multiple comparisons with Bonferroni's correction. For the effect of F0, the accuracy in “0” (69.41 ± 28.15%) was significantly higher than that in “−4” (60.66 ± 28.14%) (*p* = 0.01) and “+4” (59.27 ± 29.50%) (*p* = 0.002), and the accuracy in “+2” (65.69 ± 26.78%) was also significantly higher than that in “+4” (*p* = 0.02), indicating that performance deteriorates with the modulation of F0. For the effect of Frequency Band, the accuracy in NORMAL (81.81 ± 22.08%) was significantly higher than that in LOW (54.98 ± 26.49%) (*p* < 0.001) and HIGH (54.92 ± 27.08%) (*p* < 0.001), but no significant difference between LOW and HIGH was observed (*p* > 0.1). This suggests that both the frequencies of F1 and F2, and those of F3 and higher, significantly contribute to voice recognition. To further validate this possibility, the accuracies of SVR and OVR in LOW and HIGH were compared to the chance level (20%) by using one-sample *t*-tests. Accuracy was significantly higher than the chance level at every comparison (20 comparisons, two-tailed *p* < 0.05).

**Table 1:** **Three-way repeated measures ANOVA results**.

**Factors**	***F*-test**	***p*-value**	***Post-hoc* contrast**	***p*-value**
Identity	*F*_(1, 29)_ = 2.33	0.14		
F0^*^	*F*_(4, 116)_ = 6.53	<0.001	“−4” < “0”	0.01
			“+4” < “0”	0.002
			“+4” < “+2”	0.02
Frequency Band^*^	*F*_(2, 58)_ = 93.61	<0.001	NORMAL > LOW	<0.001
			NORMAL > HIGH	<0.001
Identity × F0	*F*_(4, 116)_ = 0.22	0.77		
Identity × Frequency Band	*F*_(2, 58)_ = 2.11	0.14		
F0 × Frequency Band^*^	*F*_(8, 232)_ = 5.30	<0.001		
Identity × F0 × Frequency Band	*F*_(8, 232)_ = 0.59	0.70		

Within-subject factors: Identity (SVR, OVR), F0 (−4, −2, 0, +2, +4 semitones) and Frequency Band (NORMAL, LOW, HIGH). The asterisks represent significant main effect or interaction.

**Figure 3:**
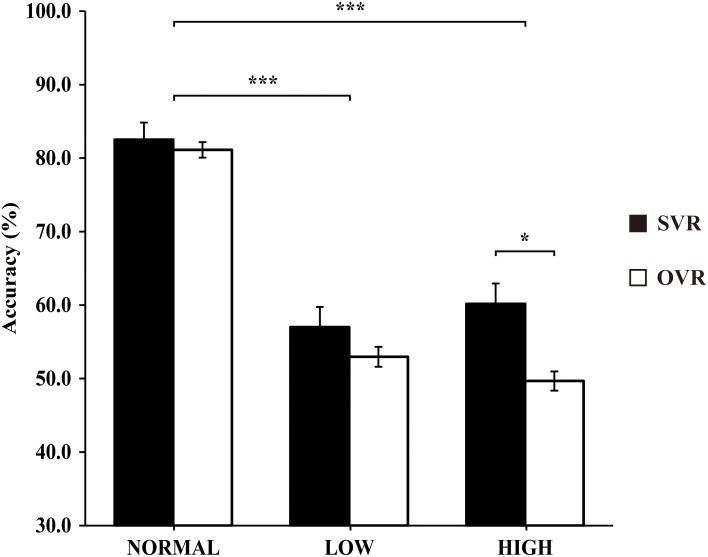
**The mean accuracies of SVR and OVR under NORMAL, LOW, and HIGH.** These mean accuracies were calculated by collapsing the accuracies of the 5 conditions (−4, −2, 0, +2, +4 semitones) of F0 modulation. The accuracies for SVR are represented by black bars, and those for OVR are represented by white bars. The error bars represent the standard error of the mean among participants. The asterisks indicate the levels of significance in the statistical analyses (^*^p < 0.05, ^***^p < 0.001). Notably, the Identity effect (SVR > OVR) is significant only in HIGH.

In order to elucidate the significant interaction of F0 × Frequency Band, follow-up two-way repeated-measures ANOVAs (within-subject factors: Identity and F0) were performed separately on the 3 levels of Frequency Band, i.e., NORMAL, LOW, and HIGH (see Table 2). A significant main effect of F0 was found in NORMAL [*F*_(4, 116)_ = 6.11, *p* = 0.001, η^2^_*p*_ = 0.17] and LOW [*F*_(4, 116)_ = 10.02, *p* < 0.001, η^2^_*p*_ = 0.26], but not in HIGH [*F*_(4, 116)_ = 0.64, *p* = 0.59]. Notably, a significant main effect of Identity was found only in HIGH [*F*_(1, 29)_ = 4.55, *p* = 0.04, η^2^_*p*_ = 0.14], with the accuracy of SVR (60.17 ± 26.67%) being significantly higher than that of OVR (49.67 ± 12.93%), but not in either NORMAL [*F*_(1, 29)_ = 0.15, *p* = 0.70] or LOW [*F*_(1, 29)_ = 0.78, *p* = 0.39] (see Table 2 and Figure 3).

**Table 2:** **Two-way repeated measures ANOVA results in NORMAL, LOW, and HIGH, respectively**.

**Conditions**	**Factors**	***F*-test**	***p*-value**	***Post-hoc* contrast**	***p*-value**
NORMAL	Identity	*F*_(1, 29)_ = 0.15	0.70		
	F0^*^	*F*_(4, 116)_ = 6.11	0.001	“−4” < “0”	0.04
				“+4” < “0”	0.003
				“+4” < “+2”	0.01
	Identity × F0	*F*_(4, 116)_ = 0.75	0.48		
LOW	Identity	*F*_(1, 29)_ = 0.78	0.39		
	F0^*^	*F*_(4, 116)_ = 10.02	<0.001	“−4” < “0”	0.002
				“−2” < “0”	0.005
				“+4” < “0”	<0.001
				“+2” < “0”	0.001
				“+4” < “+2”	0.02
	Identity × F0	*F*_(4, 116)_ = 0.12	0.88		
HIGH	Identity^*^	*F*_(1, 29)_ = 4.55	0.04	SVR > OVR	0.04
	F0	*F*_(4, 116)_ = 0.64	0.59		
	Identity × F0	*F*_(4, 116)_ = 0.38	0.73		

Within-subject factors: Identity (SVR, OVR) and F0 (−4, −2, 0, +2, +4 semitones). The asterisks represent significant main effect or interaction.

## Discussion

Self-recognition is critically involved in many circumstances, such as social interaction, in our everyday life. Together with self-face recognition, SVR is thought to play primary roles in shaping the physical aspects of self-recognition (Uddin et al., 2007; Hughes and Nicholson, 2010). The present study investigated the key acoustic cues used for SVR. The main findings are as follows: (1) compared to NORMAL, wherein the formant information is fully retained, the accuracy of SVR decreased significantly in LOW and HIGH, wherein only a specific range of frequency information was preserved. Reduced performance in these conditions was also found for OVR. (2) In NORMAL and LOW, the accuracies of SVR dropped significantly with the increment in F0 modulation, similarly to OVR. (3) In HIGH, interestingly, the accuracy of SVR was significantly higher than that of OVR, and the F0 hardly influenced the accuracy of either SVR or OVR.

With respect to result (1), the observation that the accuracies of both SVR and OVR decreased significantly as a result of the cut-off of particular frequency bands indicates that both the frequency structures of F3 and higher, and those of F1 and F2, are generally important for SVR as well as OVR. This statement is corroborated by the fact that the accuracies of both SVR and OVR in LOW and HIGH were still significantly higher than the chance level. Consistent with our results, previous studies have shown that particular formant features contain various sources of important information regarding the speaker's identity. Specifically, the F1 and F2 structures can be voluntarily altered by the speaker by changing the position of articulatory organs (e.g., tongue and jaw) (Maeda, 1990). Therefore, these formant structures may, in addition to determining the vowel features, roughly characterize the specific manner of one's speech, serving as dynamic cues for speaker identification. Moreover, the features of frequency band higher than about 2500 Hz (normally including adult formants higher than F3) have been shown to be highly dependent on the physical features of the individual vocal tract, particularly the laryngeal cavity, whose configuration almost remained invariant during one's articulation of different vowels but varied between speakers (Dang and Honda, 1997; Kitamura et al., 2005, 2006; Takemoto et al., 2006). Therefore, such higher frequencies may serve as static cues for voice recognition. In addition, our observation of reliable voice recognition in LOW is consistent with previous studies on speaker identification, which used sine-wave vocal stimuli preserving only the lowest 3 formants and found that people can recognize familiar voices by using only residual phonetic information (Fellowes et al., 1997; Remez et al., 1997).

Regarding results (2) and (3), the effects of the F0 and Identity were different in HIGH from those in NORMAL and LOW. First, the F0 significantly influenced the accuracies of both SVR and OVR in NORMAL and LOW but not in HIGH. As to NORMAL and LOW, the effect of the F0 on task performance showed a similar “inverted U-shaped” pattern with maximal performance when there was no F0 modulation (see in Figure 2 and Table 2). This observation is in line with a previous study showing a significant effect of the F0 on successful voice recognition, in which subjects were required to classify a series of F0-modulated voices into self-voice versus other-voice (Johns et al., 2006). Additionally, our observation of the significant performance difference between NORMAL and HIGH is consistent with a recent study using brief vowels, which indicated that successful voice recognition is not determined by F0 alone but by the interaction of the F0 and the formant information lower than F3 (Latinus and Belin, 2012).

By comparison, in HIGH, people could still recognize voices of their own and others, even when both the F0 and the formants lower than F3 were eliminated by high-pass filtering. Considering the “missing fundamental” theory (Licklider, 1951), which refers to the phenomenon that the pitch of a sound can be perceived from the formant structures even if it lacks the F0 component (Zatorre, 2005), one may speculate that listeners can utilize the available frequencies higher than F3 to perceive the pitch in HIGH. However, the absence of the F0 effect on task performance in HIGH makes the possible contribution of “missing fundamental” very minor, if any. Therefore, we postulate that the frequencies above F3 may mainly contribute to successful voice recognition in HIGH, even though to a reduced degree relative to in NORMAL.

Most importantly, the effect of Identity, i.e., higher accuracy in SVR than in OVR, was only observed in HIGH but neither in NORMAL nor in LOW. Frequencies above 2200 Hz have been shown to differ greatly between speakers but remain relatively constant within a speaker, allowing them to provide invariant clues for speaker identification (Li and Hughes, 1974; Kitamura and Akagi, 1995). Hence, one possible explanation to the SVR advantage in HIGH would be that the speaker has the privilege of utilizing such higher formant information of his/her own voice possibly by virtue of greater auditory familiarity with his/her own voice compared to others' voices. Access to such higher formant information may contribute to the robustness of the representation for SVR by providing stable information that is resistant to temporary acoustic variations caused by physical or emotional states. It would be interesting to examine in future studies whether a similar effect of auditory familiarity can be observed for highly familiar other-voice.

Another possible explanation for the SVR advantage in HIGH would be that the auditory representation for self-voice can be supported by its strong association with other representations of the self, such as the motor/articulatory representation (Hickok and Poeppel, 2000). In acoustically demanding situations, like the HIGH of our study, such strong association between auditory and motor/articulatory representations could compensate for the acoustic degradation and provide robust grounds for access to the higher-order representation of the self. Although auditory and motor/articulatory association could also exist for other-voice (Uddin et al., 2007), it is reasonable to assume that such association would be more robustly represented for self-voice than for other-voice, given the substantial experience of vocalizing in everyday life. In summary, the SVR advantage may be underpinned by richer self-voice representation, which is substantiated by neurocognitive factors, including auditory familiarity and cross-modal association with motor/articulatory representation.

Our results revealed that the F0 and formant structures could contribute to SVR in a distinct manner, as compared with OVR, even though some common acoustic cues are shared by SVR and OVR. While such findings are expected to provide broad implications for models of SVR, several limitations are worth noting. In view of the apparent anatomical differences in the articulatory system between males and females (e.g., the laryngeal cavity), it is necessary to further examine possible sex effects on SVR and OVR. Another limitation is that SVR was examined using off-line recorded voices, while we normally listen to our own voices during the utterance of speech. In future studies, we would like to use strategies such as the one used by Kaplan et al. (2008), who applied an equalization filter to each self-voice recording that increased frequencies below 1000 Hz by 2 dB and decreased frequencies above 1000 Hz by 2 dB to make one's own voice sound more similar to hearing it in a natural setting. Sense of execution of articulatory motor commands generates a series of neural events including collateral feed-forward signals to the auditory cortex, all of which contribute to a “sense of agency” that serves for SVR. Therefore, future studies should devise experimental setups to examine “on-line” modes of SVR while producing speech, such as the modulated auditory feedback.

## Conclusion

To summarize, we investigated the roles of the F0 and formant information in SVR by manipulating them independently. Our results revealed that the accuracies of SVR and OVR both declined as a result of either modulation of the F0 or removal of a specific formant frequency range, indicating their contributions to general voice recognition. Besides the common effects of these acoustic cues on SVR and OVR, we observed that performance of SVR was significantly better than that of OVR when only the formants higher than F3 were retained. These findings indicate partly distinct voice representation for self from that for others, which may enable generating a sense of self even under acoustically challenging situations.

### Conflict of interest statement

The authors declare that the research was conducted in the absence of any commercial or financial relationships that could be construed as a potential conflict of interest.
